# mSphere of Influence: No More Excuses—Addressing Race, Racism, and Socioeconomic Issues in the Science Classroom and Laboratory

**DOI:** 10.1128/mSphere.00010-21

**Published:** 2021-01-20

**Authors:** Pascale S. Guiton

**Affiliations:** a Department of Biological Sciences, California State University East Bay, Hayward, California, USA

**Keywords:** science and society, science education, scientist-educators, socioscientific issues, undergraduate education

## Abstract

Pascale Guiton works in the field of parasitology at a primarily undergraduate institution. In this mSphere of Influence article, she reflects on her difficulties as a faculty of color to discuss socioscientific issues in her classrooms. T. D. Sadler’s article “Situating socio-scientific issues in classrooms as a means of achieving goals of science education” (*in* T. Sadler, ed., *Socio-Scientific Issues in the Classroom. Contemporary Trends and Issues in Science Education*, vol. 39, https://doi.org/10.1007/978-94-007-1159-4_1, 2011) made an impact on her by providing her with a framework that allows her to effectively address matters of race, racism, and disparities in the context of science courses, bringing together her identity, her students’ experiences, and her perceived role as a scientist-educator. She urges scientist-educators to make real space in their curricula to address these issues.

## Abstract (precis)

Pascale Guiton works in the field of parasitology at a primarily undergraduate institution. In this mSphere of Influence article, she reflects on her difficulties as a faculty of color to discuss socioscientific issues in her classrooms. T.

## COMMENTARY

“At 2:00 pm, a train departs Paris for Rouen at 150 km/h. At 2:40 pm, a second train leaves Rouen to Paris at 120 km/h. At what time will they meet with the two cities being 130 km apart?” Easy problem, right? Not so much for an 8th grader in the Ivory Coast who had neither been on a train nor to France. I was unable to visualize the scene, panicked, and left the question unanswered. The context was too foreign for most African students. My poor performance on this assignment did not reflect my understanding or lack thereof of the relationship between speed, time, and distance. The lesson from this experience is forever ingrained in my mind: my background should not impede my ability to succeed in science. Years later, I trust that my role as a scientist-educator is to neither encourage rote learning nor alienate the learner in my classrooms and my laboratory but rather to connect them with science in a way that enriches their day-to-day lives and contributes to making them informed citizens.

I confess my course contents remain mostly out of touch with my lived experiences and that of my students. Tragically, I have unconsciously (and consciously at times) abandoned myself on the threshold of my classrooms, as I rejected my station in the gender and racial spaces and refused to be defined by my sex and the abundance of melanin in my skin. I became someone else in my workplace, a person wholly described in Paul Dunbar’s 1896 poem “We Wear a Mask” (1). Like many scientist-educators, I found it easier to justify the lack of substantive discussions of socioeconomic issues with time constraints, the sizeable instructional materials, the impact on student evaluations, and vitriolic feedback. When mentioned, these issues are often reduced to one or two bullet points on a lecture slide.

However, behind the mask hid a scientist-educator screaming about the social inequities pervading our societies and the lack of representation of people of color and women in scientific textbooks and at conferences. The mask also concealed the distress caused by pseudoscientific theories (e.g., race realism) that reinforce stereotypes and white supremacy. I became all but impervious to the struggles students face every day: homelessness, food insecurity, mental health issues, harmful stereotypes, fear of deportation, racism in all its forms, and sadly, feelings of alienation within the ivory tower. These challenges have been exacerbated by the global coronavirus disease 2019 (COVID-19) pandemic and are now laid bare for everyone to see. Why did I remain silent? What prevented me from discussing the use of scientific theories to create a logic for harm and justify harmful policies in my classroom?

In the wake of the Black Lives Matter movement and a nation reckoning with her racist history and xenophobic policies, it became impossible to hide my true self and dissociate my course contents from the social issues around me. As a scientist and college professor who happens to be a proud woman and an immigrant with a mixed racial heritage, I could no longer shy away from controversial science-related issues, including the impact of scientific research on disenfranchised communities, the stigmatization of mental illnesses and health disparities, disease transmission in immigrant detention centers and prisons, U.S. politics during a global pandemic, and the anti-vaccine movement. Anguished, yet resolute, I removed the mask, and after years of silence, I spoke freely about difficult science-related topics. The rewards were a genuine connection with my students and a renewed sense of self.

Of course, one needs not share my background or field of study to bring civic engagement into the science classroom or laboratory. Scientist-educators must only be willing to step out from behind their masks and down from the pedestals on which they sat for far too long, recognize the biases and vulnerabilities they carry into the classroom, and intentionally become change agents to achieve true inclusiveness and diversity in the science classrooms and laboratories. Nazzy Pakpour, with whom I work closely to revamp microbiology courses at our institution, accurately states “our science classrooms are critical spaces where we must challenge our students’ assumptions before releasing them into the workforce.” We can achieve this aim by addressing real issues in the classrooms and not cowering behind simple analogies, mundane experiences, or made-up case studies.

As I struggled to integrate substantive discussions of racism, poverty, democracy, and social inequities in my science lectures, I came across Troy D. Sadler’s framework for developing and implementing courses around socioscientific issues (SSI) (2). This approach offers an opportunity for scientist-educators to integrate controversial topics effectively as they transmit scientific knowledge. David Senchina’s course “Disease, Dialogue, and Democracy” is one excellent example of weaving together past and contemporary SSI with the study of historical disease outbreaks (3). Students in SSI classrooms can still engage in scientific inquiry, develop ethical decision-making skills, and improve their critical thinking and communication skills (2). I have yet to develop such a course. Still, as a scientist-educator looking to raise awareness of social issues in the science classroom, developing SSI-based lectures on commonly taught topics like gene editing, bioterrorism, stem cell research, and climate change, is as worthwhile as it is restorative.

Scientist-educators must embrace the critical responsibility to guide students toward scientific literacy that informs civic literacy. We should strive to engage students in more in-depth discussions around science-related issues, social justice, and basic human rights. It is time we join forces with our colleagues in the humanities and use our subjects to empower students as they navigate the world. With our help, science students can build the courage to address science-related issues that impact their communities and become change agents in their own right. Our students can only benefit from the truth of science and of who we are; after all, they are engaged in the pursuit of truth and knowledge.
